# Factors associated with underweight, overweight, and obesity in Chinese children aged 3–14 years using ensemble learning algorithms

**DOI:** 10.7189/jogh.15.04013

**Published:** 2025-02-07

**Authors:** Kening Chen, Fangjieyi Zheng, Xiaoqian Zhang, Qiong Wang, Zhixin Zhang, Wenquan Niu

**Affiliations:** 1 China-Japan Friendship Hospital, Chinese Academy of Medical Sciences & Peking Union Medical College, Beijing, China; 2 Center for Evidence-Based Medicine, Capital Institute of Pediatrics, Beijing, China; 3 Graduate School, Beijing University of Chinese Medicine, Beijing, China; 4 Department of Pediatrics, China-Japan Friendship Hospital, Beijing, China; 5 International Medical Services, China-Japan Friendship Hospital, Beijing, China

## Abstract

**Background:**

Factors underlying the development of childhood underweight, overweight, and obesity are not fully understood. Traditional models have drawbacks in handling large-scale, high-dimensional, and nonlinear data. In this study, we aimed to identify factors responsible for underweight, overweight, and obesity using machine learning methods among Chinese children.

**Methods:**

Our study participants were children aged 3–14 from 30 kindergartens and 26 schools in Beijing and Tangshan. Weight status was defined per the World Health Organization criteria. We implemented three ensemble learning algorithms and compared their performance and ranked the contributing factors by importance and identified an optimal set. A user-friendly web application was developed to calculate the predicted probability of childhood underweight, overweight, and obesity.

**Results:**

We analysed data from 18 503 children aged 3–14, including 1798 underweight, 10 579 of normal weight, 3257 overweight, and 2869 with obesity. Of all algorithms, random forest performed the best, with the area under the receiver operating characteristic reaching 0.759 for underweight, 0.806 for overweight, and 0.849 for obesity, with other metrics also reinforcing this algorithm. Further cumulative analyses showed that, for underweight, the optimal set of six factors included maternal body mass index (BMI), age, paternal BMI, maternal reproductive age, paternal reproductive age, and birth weight. The optimal set for overweight comprised of five factors: age, fast food intake, maternal BMI, paternal BMI, and sedentary time. For obesity, the optimal set included six factors: age, fast food intake, maternal BMI, paternal BMI, sedentary time, and maternal reproductive age. Further logistic regression analyses confirmed the predictive capability of individual top factors.

**Conclusions:**

Our findings indicate that random forest is the best ensemble learning algorithm for predicting underweight, overweight, and obesity in children aged 3–14 years. We identified the optimal set of significant factors for each malnutrition status and incorporated them into a web application to support the application of this study’s findings.

Malnutrition is a global public health threat and is the cause behind nearly 50% of all deaths of children under five years [1]. Its burden revolves around either undernutrition or overnutrition. Statistics from the United Nations Children’s Fund and World Health Organization (WHO) show that 6.7% of children under five years worldwide and 3.6% in China specifically are affected by wasting [2,3]. Yet simultaneously, health systems worldwide are tackling the childhood obesity epidemic [4]. According to the WHO, the number of children aged 5–19 years with obesity has risen from 11 million in 1975 to 340 million in 2023 [5]. In China, the prevalence of overweight and obesity was estimated to be 6.8% and 3.6% in children under six years, and 11.1% and 7.9% in children aged 6–17 years, respectively [6]. Evidence shows that underweight, overweight, and obesity negatively impact children’s health, and a deeper understanding of potential risk factors behind malnutrition can enhance our knowledge of weight management and support the development of effective prevention strategies.

The development of childhood underweight, overweight, and obesity is a complex process to which inherited and non-inherited factors contribute individually and interactively, including parental obesity [7], gestational weight gain [8], and lifestyle habits [9]. However, findings from such studies are often not reproducible, with no consensus on their implications. For example, some studies have reported a significant association between late bedtime and obesity in children [10–12], while others reported contrasting findings [13,14]. These inconsistencies could be due to observations being multifaceted, possibly because of different body builds, miscellaneous nutritional habits, various diagnostic criteria, and diverse analytical methodologies. To date, most studies on this subject have been cross-sectional. Although case-control studies cannot replace cohort studies in identifying causal factors associated with abnormal weight in children, they are an important alternative strategy, especially in the context of sufficient sample sizes, ethnically homogeneous populations, internationally accepted diagnostic criteria, and advanced statistical methodologies.

With this in mind, we aimed to test the hypothesis that factors selected and compiled by machine learning algorithms can significantly predict the risk for underweight, overweight, and obesity.

## METHODS

### Study design and participants

We conducted two large cross-sectional surveys in 2020 and 2022 using random sampling techniques, collecting demographic and health-relevant data from children aged 3–14 from schools in Beijing and Tangshan.

Specifically, we collected our data in two waves using stratified cluster sampling methods. The first collection wave lasted from September to December 2020, with preschool-aged children being sampled from four out of sixteen districts in Beijing and two out of seven districts in Tangshan. Five kindergartens were selected within each district for a total of 30. The second wave occurred in January 2022 in Pinggu District, Beijing, among eight primary and 18 junior high schools.

### Data collection and variable definition

At baseline, physical assessments included standardised measurements of height (in centimeters, rounded to the nearest single decimal place) and weight (in kilograms, rounded to the nearest single decimal place) carried out by health practitioners in selected kindergartens and schools. The same measuring equipment was distributed to each kindergarten or school, with the health practitioners and teachers in charge being trained to ensure that measurement methods, procedures, readings, and biases were as consistent as possible.

We used structured questionnaires to collect information on five dimensions: demographic characteristics (date of birth, age, sex, height, and weight), fetal and early life factors (gestational week, mode of delivery, pregnancy and delivery order, twin birth, length and weight at birth, infancy feeding, breastfeeding duration, and time to add solid food), lifestyle-related factors (sedentary time, screen time, time of outdoor activities, bedtime, eating speed and weekly intake frequencies of sweet food, night meals and fast food), health status (food or drug allergies, number of dental caries, and chronic illnesses), and family information (family income, parental height and weight, parental education level, and parental reproductive age) (Table S1 in the **Online Supplementary Document**). We selected all variables based on expert knowledge and available literature on potential factors responsible for childhood underweight, overweight and obesity, to identify suspected or established factors associated with malnutrition.

We assessed the reliability and validity of self-designed questionnaires by initially distributing 200 samples before formal distributions. The reliability coefficient (α) was over 0.85 for both questionnaires.

### Quality control

Prior to data collection, health practitioners and teachers-in-charge from selected kindergartens and schools were trained about survey procedures and items in questionnaires, and they assisted parents or guardians of participating children during surveys. The teachers contacted the parents or guardians in case of missing data or abnormal values.

### Definition of weight metrics

Body mass index (BMI) was calculated as weight in kilograms divided by height in meters squared (kg/m^2^). We used children’s age- and sex-standardised BMI z-scores to classify the different weight status categories, which we had calculated as the deviation of an individual’s BMI from the population mean, expressed in standard deviations (SDs). Based on the 2006 WHO growth standards for children aged 0–5 years [15] and the 2007 WHO growth reference for school-aged children aged 5–19 years (61–228 months) [16], we defined underweight as a BMI z-score<−2 SD, overweight as a BMI z-score>+1 SD, and obesity as a BMI z-score>+2 SD among children.

### Statistical analyses

We used R, version 4.3.2 (R Core Team, Vienna, Austria) for our statistical analyses. We removed variables with over 30% missing data from analyses and used the multiple imputation method for variables with less than 30% missing data. After filling in missing data using the ‘R-MICE’ package, we applied propensity score matching to balance sex between children with normal weight and those classified as underweight, overweight, or obese. We randomly divided the data into the training set (70%) and testing set (30%) for cross-validation. We then conducted a between-group comparison using the *t*-test for normally distributed continuous variables (expressed as means and standard deviations (SDs)), the rank sum test for skewed continuous variables (expressed as medians and interquartile ranges (IQR)), and the χ^2^ (χ^2^) test for categorical variables (expressed as counts and percentages).

We employed logistic regression and three ensemble learning algorithms – decision tree, random forest, and gradient boosting machine – to model variables under investigation associated with childhood underweight, overweight, and obesity. There were advantages and disadvantages to all three ensemble learning algorithms, and the impact of hyperparameter tuning on the performance of these algorithms varied (Tables S2 and S3 in the **Online Supplementary Document**). We assessed the optimal algorithm with the best performance by accuracy (*i.e.* prediction of correct outcomes as a percentage of the total sample), Brier score (*i.e.* the mean of the squared difference between the observed and predicted event rates), area under the receiver operating characteristic curve (AUROC) (derived from plotting sensitivity vs. 1-specificity), and decision curve analysis (for estimating the net benefit of a predictive model), as well as the mean absolute error, mean squared error, area over the regression error characteristic curve, and area over the regression receiver operating characteristic. We selected the algorithm with the best overall performance across these metrics. Additionally, under the optimal algorithm, we assessed the importance of factors under investigation and ranked them in descending order for the prediction of underweight, overweight, and obesity. We determined the optimal number of top factors by evaluating cumulative performance across AUROC, accuracy, and precision. Generally, with the addition of important factors, these metrics gradually improve until plateauing, which then forms the optimal set of features. Furthermore, to facilitate the interpretation of factors finally determined via the optimal algorithm, we implemented a logistic regression model for underweight, overweight, and obesity, and expressed effect-size estimates as odds ratio (OR) and 95% confidence interval (CI). Finally, we developed a user-friendly Shiny web application to calculate the predicted probability of childhood underweight, overweight, and obesity. We analysed the data between January and March 2024.

### Ethical considerations

This study included two waves, and we separately obtained ethical approval from the Ethics Committee of the China-Japan Friendship Hospital and Beijing University of Chinese Medicine. The parents or guardians of the enrolled children provided written informed consent. Data collection procedures adhered to local data protection regulations. We securely stored all data on an access-restricted server, accessible only to our research team members, and we anonymised personally identifiable information by assigning unique identifiers before analyses, ensuring the confidentiality of participants’ identities. We followed the STROBE guidelines in reporting our findings (Table S4 in the **Online Supplementary Document**).

## RESULTS

### Baseline characteristics

**Figure 1:**
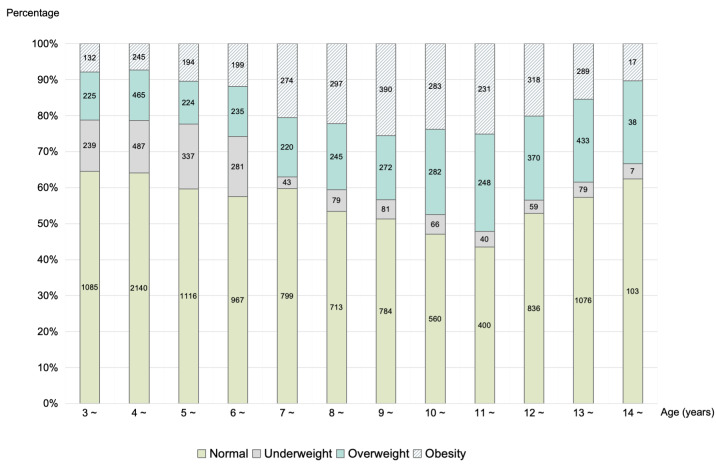
BMI categories subdivided into age groups of study children. Weight status was defined according to the criteria recommended by the WHO [15,16]. BMI – body mass index.

We enrolled 18 503 children aged 3–14 years from 30 kindergartens and 26 primary and junior high schools in Beijing and Tangshan, of whom 10 579 were of normal weight, 3257 were overweight, 1798 were underweight, and 2869 had obesity (**Figure 1**, **Table 1****;** Figure S1 in the **Online Supplementary Document**).

**Table 1:** Baseline characteristics of study children by weight status

		Weight status*			
	**All participants, (n = 18 503)**	**Underweight, (n = 1798)**	**Normal weight, (n = 10 579)**	**Overweight, (n = 3257)**	**Obesity, (n = 2869)**
**Demographic characteristics**					
Age (months)†	90 (58–131)	66 (52–85)	83 (56–127)	108 (66–145)	110 (77–139)
Boys‡	9522 (51.5)	938 (52.2)	4835 (45.7)	1734 (53.2)	2015 (70.2)
**Foetal and early life factors‡**					
Gestational age in weeks	39 (38–40)	39 (38–40)	39 (38–40)	39 (38–40)	39 (38–40)
Full-term birth‡					
*Preterm delivery*	1757 (9.5)	172 (9.6)	975 (9.2)	300 (9.2)	310 (10.8)
*Normal delivery*	16 181 (87.5)	1562 (86.9)	9276 (87.7)	2870 (88.1)	2473 (86.2)
*Post-term pregnancy*	565 (3.1)	64 (3.6)	328 (3.1)	87 (2.7)	86 (3.0)
Delivery mode‡					
*Vaginal delivery*	9130 (49.3)	939 (52.2)	5533 (52.3)	1451 (44.6)	1207 (42.1)
*Caesarean section*	9329 (50.4)	853 (47.4)	5024 (47.5)	1794 (55.1)	1658 (57.8)
*Forceps delivery*	44 (0.2)	6 (0.3)	22 (0.2)	12 (0.4)	4 (0.1)
Pregnancy order†	1 (1–2)	2 (1–2)	1 (1–2)	1 (1–2)	1 (1–2)
Delivery order†	1 (1–2)	1 (1–2)	1 (1–2)	1 (1–1)	1 (1–1)
Twin birth‡	445 (2.4)	39 (2.2)	274 (2.6)	71 (2.2)	61 (2.1)
Birth length in cm†	50 (50–52)	50 (50–52)	50 (50–52)	50 (50–52)	50 (50–52)
Birth weight in kg†	3.35 (3.00–3.60)	3.30 (3.00–3.60)	3.30 (3.00–3.60)	3.40 (3.10–3.70)	3.50 (3.10–3.75)
Infancy feeding‡					
*Pure breastfeeding*	10 501 (56.8)	1060 (59.0)	6046 (57.2)	1813 (55.7)	1582 (55.1)
*Partial breastfeeding*	6353 (34.3)	599 (33.3)	3624 (34.3)	1163 (35.7)	967 (33.7)
*Non-breastfeeding*	1649 (8.9)	139 (7.7)	909 (8.6)	281 (8.6)	320 (11.2)
Breastfeeding duration‡					
*<6 mo*	5475 (29.6)	372 (20.7)	2970 (28.1)	1067 (32.8)	1066 (37.2)
*6–24 mo*	11 248 (60.8)	1182 (65.7)	6546 (61.9)	1962 (60.2)	1558 (54.3)
*≥24 mo*	1780 (9.6)	244 (13.6)	1063 (10.0)	228 (7.0)	245 (8.5)
Time to add solid food‡					
*<6 mo*	3260 (31.6)	321 (34.5)	1830 (32.2)	598 (31.0)	511 (28.8)
*6–9 mo*	5327 (51.7)	433 (46.5)	2930 (51.6)	1013 (52.5)	951 (53.5)
*≥9 mo*	1725 (16.7)	177 (19.0)	916 (16.1)	317 (16.4)	315 (17.7)
**Lifestyle-related factors†**					
Sedentary time (hours per day)	3.86 (2.00–6.29)	2.57 (1.29–4.86)	3.57 (1.71–6.00)	4.43 (2.00–6.71)	4.86 (2.29–6.86)
Screen time (hours per day)	1.00 (0.64–1.57)	1.00 (0.64–1.57)	1.00 (0.64–1.57)	1.29 (0.71–1.71)	1.29 (0.93–2.00)
Outdoor activities (hours per day)	1.29 (1.00–2.00)	1.29 (1.00–2.00)	1.29 (1.00–2.00)	1.29 (1.00–2.00)	1.29 (1.00–2.00)
Bedtime (o’clock PM)	9.50 (9.00–10.00)	9.00 (9.00–10.00)	9.50 (9.00–10.00)	10.00 (9.00–10.00)	10.00 (9.00–10.00)
Eating speed (minutes per meal)	16.67 (13.33–23.33)	18.33 (15.00–25.00)	18.33 (13.33–23.33)	16.67 (13.33–20.00)	16.67 (13.33–20.00)
Fast food intake frequency‡					
*Every day*	5636 (30.5)	885 (49.2)	3524 (33.3)	760 (23.3)	467 (16.3)
*3–5 times weekly*	3230 (17.5)	481 (26.8)	1902 (18.0)	447 (13.7)	400 (13.9)
*1–2 times weekly*	5038 (27.2)	220 (12.2)	2653 (25.1)	1065 (32.7)	1100 (38.3)
*None or once in a while*	4599 (24.9)	212 (11.8)	2500 (23.6)	985 (30.2)	902 (31.4)
Sweet food intake frequency‡					
*Every day*	1699 (9.2)	207 (11.5)	1036 (9.8)	249 (7.6)	207 (7.2)
*3–5 times weekly*	6313 (34.1)	846 (47.1)	3744 (35.4)	964 (29.6)	759 (26.5)
*1–2 times weekly*	7941 (42.9)	571 (31.8)	4443 (42.0)	1521 (46.7)	1406 (49.0)
*None or once in a while*	2550 (13.8)	174 (9.7)	1356 (12.8)	523 (16.1)	497 (17.3)
Night meal intake frequency‡					
*Every day*	6047 (32.7)	312 (17.4)	3231 (30.5)	1270 (39.0)	1234 (43.0)
*3–5 times weekly*	3702 (20.0)	268 (14.9)	2033 (19.2)	698 (21.4)	703 (24.5)
*1–2 times weekly*	2952 (16.0)	368 (20.5)	1753 (16.6)	479 (14.7)	352 (12.3)
*None or once in a while*	5802 (31.4)	850 (47.3)	3562 (33.7)	810 (24.9)	580 (20.2)
**Health status**					
Food allergy‡	1897 (10.3)	213 (11.8)	1045 (9.9)	312 (9.6)	327 (11.4)
Drug allergy‡	775 (4.2)	74 (4.1)	436 (4.1)	123 (3.8)	142 (4.9)
Dental caries†	0 (0–2)	0 (0–2)	1 (0–2)	0 (0–2)	0 (0–2)
**Family information†**					
Maternal reproductive age in years	27.58 (25.25–30.33)	28.75 (26.25–32.06)	27.67 (25.42–30.42)	27.17 (24.92–29.75)	27.00 (24.67–29.50)
Paternal reproductive age in years	28.58 (26.25–31.75)	30.09 (27.25–33.67)	28.59 (26.42–31.83)	28.17 (26.01–31.17)	28.17 (25.84–30.75)
Maternal BMI in kg/m^2^	22.66 (20.51–25.39)	20.04 (18.20–22.53)	22.46 (20.43–24.97)	23.44 (21.48–26.37)	24.22 (21.88–28.04)
Paternal BMI in kg/m^2^	25.74 (23.39–28.41)	25.09 (22.86–27.77)	25.25 (23.05–27.76)	26.12 (24.05–29.38)	27.04 (24.62–30.42)
Maternal education level‡					
*High school degree or below*	7366 (39.8)	552 (30.7)	4191 (39.6)	1364 (41.9)	1259 (43.9)
*Bachelor’s degree*	10 058 (54.4)	937 (52.1)	5812 (54.9)	1763 (54.1)	1546 (53.9)
*Master’s degree or above*	1079 (5.8)	309 (17.2)	576 (5.4)	130 (4.0)	64 (2.2)
Paternal education level‡					
*High school degree or below*	8447 (45.7)	635 (35.3)	4793 (45.3)	1562 (48.0)	1457 (50.8)
*Bachelor’s degree*	8832 (47.7)	816 (45.4)	5142 (48.6)	1546 (47.5)	1328 (46.3)
*Master’s degree or above*	1224 (6.6)	347 (19.3)	644 (6.1)	149 (4.6)	84 (2.9)
Family income in CNY per year‡					
*<100 000*	7695 (41.6)	597 (33.2)	4436 (41.9)	1388 (42.6)	1274 (44.4)
*100 000–300 000*	7909 (42.7)	679 (37.8)	4521 (42.7)	1425 (43.8)	1284 (44.8)
*≥300 000*	2899 (15.7)	522 (29.0)	1622 (15.3)	444 (13.6)	311 (10.8)

BMI – body mass index, mo – months

*Weight status was defined according to the criteria recommended by the World Health Organization [15,16].

†Continuous variables presented as median (interquartile range).

‡Categorical variables presented as numbers (%).

### Selection of optimal algorithm

**Figure 2:**
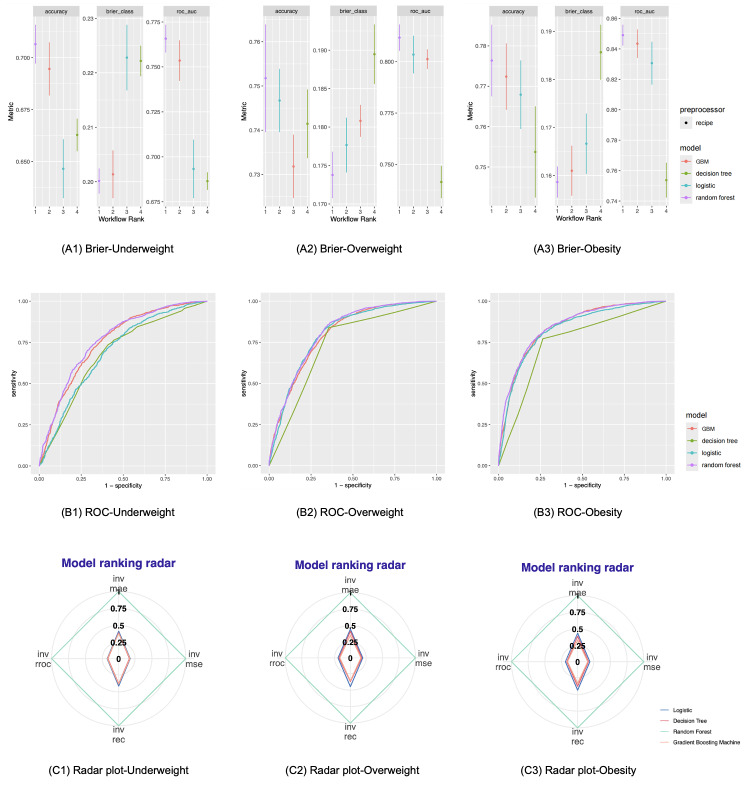
Predictive performance of 3 ensemble learning algorithms annexed with Logistic regression for childhood weight status. Weight status was defined according to the criteria recommended by the WHO [15,16]. GBM – gradient boosting machine, ROC – the receiver operating characteristic curve.

Of all algorithms, random forest performed the best, with the AUROC reaching 0.759 for underweight, 0.806 for overweight, and 0.849 for obesity (**Figure 2**). This was also confirmed statistically by other assessment metrics and visually by the decision curve analysis (Figure S2 in the **Online Supplementary Document**). We therefore selected the random forest as the optimal algorithm for the prediction of underweight, overweight, and obesity in children aged 3–14 years.

### Determination of optimal important factors

**Figure 3:**
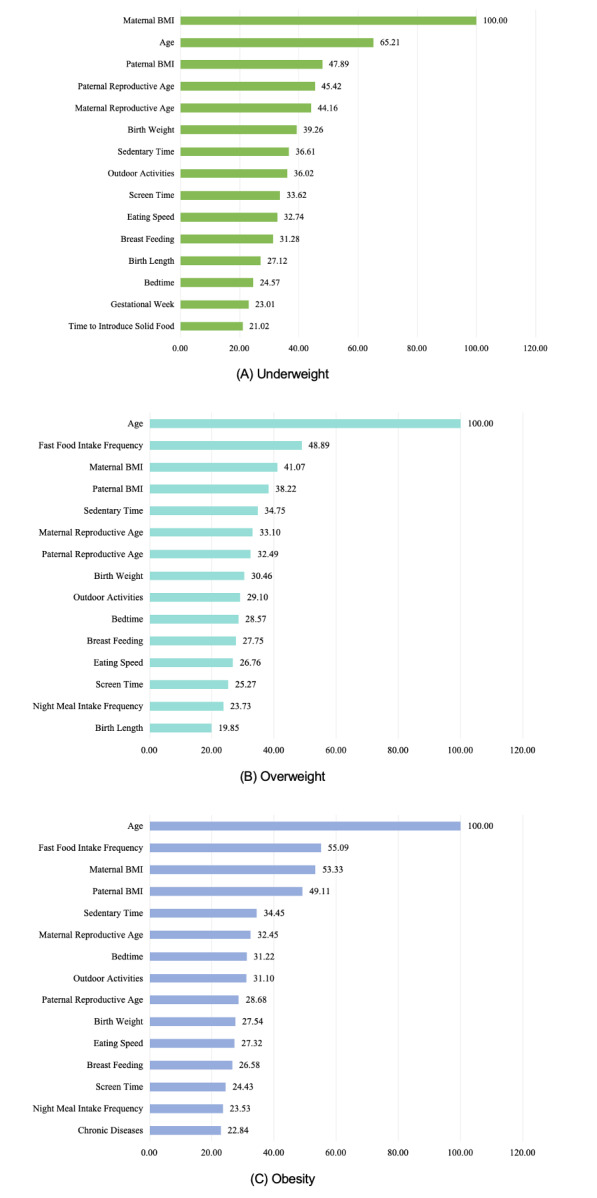
The ranking of the 15 most important variables related to childhood weight status based on the random forest. Weight status was defined according to the criteria recommended by the WHO [15,16].

Under the use of the optimal random forest algorithm, we explored the importance of the top 15 factors for childhood underweight, overweight, and obesity (**Figure 3**). To determine the optimal set of important factors, we assessed the cumulative performance of top factors using AUROC, accuracy, and precision (**Table 2**). After inspecting the changes in these indicators with the increment of important factors, we found that the cumulative performance of all three outcomes tended to increase and then decrease.

**Table 2:** AUROC, accuracy, and precision with cumulating number of top 15 factors using random forest algorithm for childhood weight status*

	Cumulating number of top 15 factors	AUROC	Accuracy	Precision
**Underweight**				
Maternal BMI	1	0.692	0.644	0.703
Age	2	0.711	0.651	0.675
Paternal BMI	3	0.749	0.688	0.701
Paternal reproductive age	4	0.740	0.675	0.695
Maternal reproductive age	5	0.747	0.673	0.692
Birth weight	6	0.753	0.690	0.705
Sedentary time	7	0.749	0.693	0.710
Outdoor activities	8	0.737	0.680	0.687
Screen time	9	0.739	0.677	0.688
Eating speed	10	0.736	0.666	0.672
Breastfeeding	11	0.741	0.679	0.685
Birth length	12	0.735	0.670	0.675
Bedtime	13	0.750	0.690	0.700
Gestational week	14	0.754	0.693	0.706
Time to add solid food	15	0.747	0.684	0.694
**Overweight**				
Age	1	0.753	0.741	0.785
Fast food intake frequency	2	0.749	0.736	0.776
Maternal BMI	3	0.757	0.727	0.765
Paternal BMI	4	0.764	0.725	0.749
Sedentary time	5	0.775	0.726	0.749
Maternal reproductive age	6	0.774	0.722	0.749
Paternal reproductive age	7	0.777	0.729	0.759
Birth weight	8	0.788	0.724	0.751
Outdoor activities	9	0.788	0.732	0.762
Bedtime	10	0.795	0.735	0.776
Breastfeeding	11	0.799	0.734	0.778
Eating speed	12	0.805	0.736	0.780
Screen time	13	0.803	0.737	0.780
Night meal intake frequency	14	0.799	0.734	0.778
Birth length	15	0.804	0.744	0.789
**Obesity**				
Age	1	0.773	0.769	0.786
Fast food intake frequency	2	0.784	0.772	0.776
Maternal BMI	3	0.808	0.755	0.761
Paternal BMI	4	0.828	0.771	0.779
Sedentary time	5	0.829	0.771	0.781
Maternal reproductive age	6	0.832	0.771	0.779
Bedtime	7	0.835	0.763	0.779
Outdoor activities	8	0.835	0.770	0.790
Paternal reproductive age	9	0.837	0.776	0.795
Birth weight	10	0.836	0.779	0.789
Eating speed	11	0.844	0.780	0.793
Breastfeeding	12	0.842	0.782	0.797
Screen time	13	0.845	0.779	0.792
Night meal intake frequency	14	0.843	0.778	0.791
Chronic diseases	15	0.844	0.779	0.794

AUROC – area under the receiver operating characteristic curve, BMI – body mass index

*Weight status was defined according to the criteria recommended by the World Health Organization [15,16].

For underweight, the optimal set of six factors included maternal BMI, age, paternal BMI, maternal reproductive age, paternal reproductive age, and birth weight; for overweight, the optimal set comprised five factors: age, fast food intake frequency, maternal BMI, paternal BMI, and sedentary time; for obesity, the optimal set included six factors: age, fast food intake frequency, maternal BMI, paternal BMI, sedentary time, and maternal reproductive age.

### Risk quantification

We used logistic regression to improve the clinical applicability of the optimal sets of important factors. Each factor in the three optimal sets was significantly associated with the risk of underweight, overweight, or obesity in children aged 3–14 years, at a significance level of 0.1% (**Table 3**).

**Table 3:** The risk prediction of top factors for childhood weight status using the logistic regression model*

	OR (95% CI)†	*P*-value
**Underweight (six factors)**		
Maternal BMI		
*Underweight (BMI < 18.5 kg/m^2^)*	5.084 (4.459–5.796)	<0.001
*Normal (BMI 18.5–25 kg/m^2^)*	ref	
*Overweight or obesity (BMI ≥ 25 kg/m^2^)*	0.579 (0.485–0.691)	<0.001
Age		
*Pre-school children (3–6 years)*	ref	
*School-age children (7–14 years)*	0.340 (0.304–0.381)	<0.001
Paternal BMI		
*Underweight (BMI < 18.5 kg/m^2^)*	3.047 (2.358–3.936)	<0.001
*Normal (BMI 18.5–25 kg/m^2^)*	ref	
*Overweight or obesity (BMI ≥ 25 kg/m^2^)*	1.016 (0.909–1.135)	0.781
Paternal reproductive age in years‡		
*24–28*	ref	
*<24 or ≥28*	1.692 (1.499–1.886)	<0.001
Maternal reproductive age in years‡		
*21–27*	ref	
*<21 or ≥27*	1.607 (1.441–1.792)	<0.001
Birth weight		
*Normal (≥2.5 kg)*	ref	
*Low birth weight (<2.5 kg)*	1.522 (1.188–1.950)	0.001
**Overweight (five factors)**		
Age		
*Pre-school children (3–6 years)*	ref	
*School-age children (7–14 years)*	1.848 (1.703–2.004)	<0.001
Fast food intake frequency		
*None or once in a while*	ref	
*1–2 times weekly*	1.090 (0.957–1.240)	0.193
*3–5 times weekly*	1.861 (1.674–1.240)	<0.001
*Every day*	1.827 (1.641–2.034)	<0.001
Maternal BMI		
*Underweight (BMI < 18.5 kg/m^2^)*	0.471 (0.377–0.589)	<0.001
*Normal (BMI 18.5–25 kg/m^2^)*	ref	
*Overweight or obesity (BMI ≥ 25 kg/m^2^)*	1.507 (1.368–1.661)	<0.001
Paternal BMI		
*Underweight (BMI < 18.5 kg/m^2^)*	1.191 (0.877–1.618)	0.263
*Normal (BMI 18.5–25 kg/m^2^)*	ref	
*Overweight or obesity (BMI ≥ 25 kg/m^2^)*	1.400 (1.281–1.529)	<0.001
Sedentary time		
*<2 h per day*	ref	
*≥2 h per day*	1.223 (1.114–1.344)	<0.001
**Obesity (six factors)**		
Age		
*Pre-school children (3–6 years)*	ref	
*School-age children (7–14 years)*	2.745 (2.506–3.006)	<0.001
Fast food intake frequency		
*None or once in a while*	ref	
*1–2 times weekly*	1.587 (1.373–1.834)	<0.001
*3–5 times weekly*	3.129 (2.777–3.526)	<0.001
*Every day*	2.723 (2.408–3.079)	<0.001
Maternal BMI		
*Underweight (BMI < 18.5 kg/m^2^)*	0.557 (0.439–0.706)	<0.001
*Normal (BMI 18.5–25 kg/m^2^)*	ref	
*Overweight or obesity (BMI ≥ 25 kg/m^2^)*	2.013 (1.819–0.706)	<0.001
Paternal BMI		
*Underweight (BMI < 18.5 kg/m^2^)*	1.057 (0.725–1.540)	0.774
*Normal (BMI 18.5–25 kg/m^2^)*	ref	
*Overweight or obesity (BMI ≥ 25 kg/m^2^)*	1.894 (1.716–1.540)	<0.001
Sedentary time		
*<2 h per day*	ref	
*≥2 h per day*	1.415 (1.278–1.567)	<0.001
Maternal reproductive age in years‡		
*27–40*	ref	
*<27 or ≥40*	1.389 (1.279–1.509)	<0.001

BMI – body mass index, CI – confidence interval, OR – odds ratio, ref – reference

*Weight status was defined according to the criteria recommended by the World Health Organization [15,16].

†ORs were adjusted for age, sex, education, and family income.

‡Subgroup thresholds for parental reproductive age are derived from the restricted cubic spline curves.

### Convenient application for clinical utility

**Figure 4:**
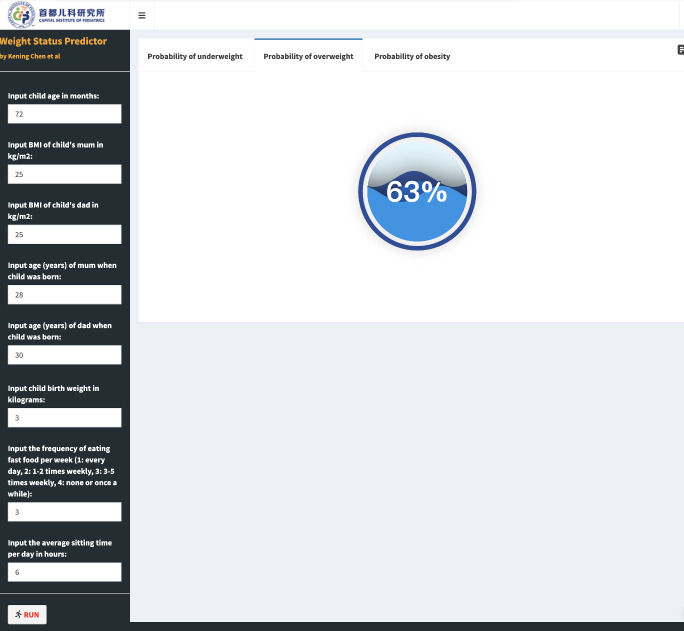
The web application [17] for the probability of childhood underweight, overweight, and obesity.

We implemented the final prediction model into a web application to enhance its usability in clinical settings (**Figure 4**). By entering the actual values required for the optimal algorithm, the application automatically calculates the probability of being underweight, overweight, or obese [17].

## DISCUSSION

We aimed to identify factors significantly associated with childhood underweight, overweight, and obesity by using machine learning algorithms among children aged 3–14 years from Beijing and Tangshan. Through comprehensive exploration, random forest consistently outperformed the other algorithms in predicting the three weight metrics, achieving an accuracy of over 75%. Moreover, the optimal set of important factors demonstrated a comparable performance to all factors assessed. To our knowledge, this is the first study to use artificial intelligence techniques to identify risk profiles for underweight, overweight, and obesity in Chinese children.

Abnormal body weight is highly prevalent in both adults and children. In most cases, childhood obesity often persists into adulthood [18,19], which has led to increased attention on identifying the factors responsible for overweight and obesity in children. At the same time, childhood underweight remains a major public health issue, with our results showing that approximately one in ten children are underweight. Despite over two decades of intensive research aimed at identifying factors contributing to abnormal weight status, there is no clear consensus on the number and specific factors involved in the predisposition to underweight, overweight, or obesity in children. A major challenge in defining the risk profiles for abnormal weight is statistical methodology, especially with the rise of artificial intelligence, which is set to revolutionise medicinal practice, improving the efficiency and accuracy of disease diagnosis [20,21]. To gain deeper insights, we employed three ensemble learning algorithms, alongside the traditional logistic regression model, to determine which algorithm performed best and to identify the minimal set of factors sufficient to predict childhood underweight, overweight, and obesity.

After a comprehensive comparison of various performance metrics, random forest consistently outperformed the others in predicting childhood underweight, overweight, and obesity. For instance, in terms of accuracy and AUROC, random forest showed superior performance compared to decision tree and gradient boosting machine algorithms, while decision tree ranked highest for Brier’s score across all three weight categories. While the AUROC is a commonly used metric for evaluating the performance of classification models, providing a thorough assessment of a model’s sensitivity and specificity trade-offs at different thresholds, evidence suggests that Brier score may be misleading in case of imbalanced data sets, as it might reflect good overall performance, but fail to capture poor performance in the minority class [22]. Moreover, random forest has been widely adopted in the literature due to its ability to improve prediction accuracy by combining a pre-specified number of decision trees and its effectiveness at handling high-dimensional data with numerous features and reducing the risk of overfitting by constructing multiple decision trees and averaging their results [23–27]. During the training process, each decision tree in a random forest considers only a random subset of the data, allowing the algorithm to capture various combinations of features, which improves generalisation ability, stability, and robustness. Additionally, random forest can be easily parallelised, meaning that multi-core processors or distributed computing resources can be used to speed up the training process when working with large data sets. These advantages have led to random forest being widely adopted in clinical settings.

Using random forest, we identified a minimum set of contributing factors for childhood underweight, overweight, and obesity, with comparable prediction performance to models that included all factors under investigation. Notably, three factors – child’s age, parental BMI, and maternal reproductive age – were commonly associated with the risk of underweight, overweight, and obesity in children aged 3–14 years. Additionally, birthweight was exclusively associated with underweight, while fast food intake frequency and sedentary time were specifically linked to overweight and obesity. Maternal reproductive age was found to be exclusively associated with childhood obesity compared to overweight. Consistent with previous studies, parental weight status appears to influence that of their children [28–31]. Research shows that a child with one obese parent is three times more likely to become obese in adulthood, and if both parents are obese, the child’s risk increases 10-fold [32]. Conversely, the risk of being underweight is higher in children with thin parents [33]. Regarding parental reproductive age, we used restricted cubic spline curves in our logistic regression analysis to determine cut-off values, showing that both very high or very low maternal age at childbearing were associated with suboptimal weight status in children. Similarly, inappropriate childbearing age was reported to increase the risk of overweight and obesity in children and interact with parental weight status [34]. Our findings suggest that the risk of overweight and obesity in offspring generally increases and then decreases with parental reproductive age, with the highest risk observed in fathers aged 24–30 years and mothers aged 24–28 years, as confirmed by our logistic regression results. In addition, variations in risk profiles for different weight statuses were found, particularly for childhood underweight, and the prevalence of malnutrition in children with low birth weight was significantly higher than in those with normal birth weight [35]. For childhood overweight and obesity, frequent fast food consumption [36] and long sedentary hours [37,38] are well-established risk factors.

In short, the most important risk factors identified in this study aligned with those found in prior studies, demonstrating the interpretability, accuracy, and robustness of our findings. Furthermore, by employing machine learning techniques, we were able to synthesise data on early childhood factors, lifestyles, and family conditions. We also ranked the contribution of relevant factors and developed a prediction tool based on a minimal set of contributing factors. This extends previous research and offers valuable insights for clinical decision-making and individualised prevention and intervention strategies for children at risk for unhealthy weight status, which are of critical importance for public health.

### Limitations

This study has several limitations. First, while BMI is an important indicator recommended by the WHO for assessing nutrition status, other body size indices, such as waist-to-hip ratio, waist-to-height ratio, and body roundness index [39], may better reflect fat distributions. However, data on waist and hip circumference were not available for this study. Second, due to the lack of uniform standards for weight status in Chinese children under five years, we applied the WHO-recommended BMI z-score thresholds to define underweight, overweight, and obesity in children. Whether this criterion is appropriate for Chinese children remains largely unknown. Third, the causes of suboptimal weight status in children are multifactorial, but only 31 factors were included in the machine learning algorithms. Most factors in this study were based on self-reported questionnaires, without the inclusion of biochemical markers, limiting the possibility for further analyses of mediating factors or mechanisms. Finally, all participants were sourced from Chinese children in Beijing and Tangshan, so the generalisability of our findings to other countries, regions, and ethnicities is limited due to the absence of external validation.

## CONCLUSIONS

Our findings suggest that random forest is the best ensemble learning algorithm for predicting underweight, overweight, and obesity in children aged 3–14 years. We identified the optimal set of significant factors for each weight status and compiled these into a web application to facilitate the broader application of this study. Our findings provide insights into the risk profiles associated with suboptimal weight status in Chinese children and highlight the potential clinical applicability of the developed model in identifying high-risk children with abnormal weight status.

## Additional material


Online Supplementary Document
