# Supplementary material for: Factors associated with underweight, overweight, and obesity in Chinese children aged 3–14 years using ensemble learning algorithms
Source: J Glob Health. 2025 Feb 7;15:04013. doi: 10.7189/jogh.15.04013 (PMC11804908; doi:10.7189/jogh.15.04013)
Supplement: Online Supplementary Document [file jogh-15-04013-s001.pdf]

Supplement to: Chen K, Zheng F, Zhang X, Wang Q, Zhang Z, Niu W. Factors associated with underweight, overweight and obesity in Chinese children aged 3–14 years using ensemble learning algorithms. J Glob Health. 2025;15:04013.

Table S1. Resource and definition of variables under study

Table S2. The comparison of 3 ensemble learning algorithms adopted in this study

Table S3. Hyper-parameters of 3 ensemble learning algorithms adopted in this study

Table S4. STROBE Statement—checklist of items that should be included in reports of observational studies

Figure S1. Selection flowchart of study participants

Figure S2. Decision curve analysis of 3 machine learning algorithms annexed with Logistic regression for childhood weight status

Table S1. Resource and definition of variables under study

Variables	Questionnaire questions or definition	Categories
**Demographic Characteristics**		
Age	What is the date of birth of your child?	We converted the children's date of birth to age in months from the date of the survey (to the nearest 1 month)
Sex	What is the sex of your child?	Female, Male
Height	The health practitioner at the target school took a standardized measurement of the children's height.	Continuous (to the nearest 0.1 cm)
Weight	The health practitioner at the target school took a standardized measurement of the children's weight.	Continuous (to the nearest 0.1 kg)
Body mass index (BMI)	BMI was calculated as weight in kilograms divided by height in meters squared (to the nearest 0.01 kg/m^2).	Based on the 2006 WHO-defined growth standards for children aged 0-5 years (0-60 months) and the 2007 WHO growth reference for school-age children and adolescents aged 5-19 years (61-228 months), underweight was defined as BMI z-score < - 2 SD, overweight was defined as BMI z-score > +1 SD, and obesity was defined as BMI z-score > + 2 SD among children.

**Foetal and Early Life Factors**		
Gestational age	What was the gestational age of your child at birth? (to the nearest 1 week).	According to the World Health Organization (WHO) recommendations, we classified the gestational weeks into three groups to identify whether the child was born at full term or not: (1) Preterm delivery (those who reported $<$ 37 weeks of gestation); (2) Normal (those who reported $\geq$ 37 weeks to $<$ 42 weeks of gestation); and (3) Postterm pregnancy births (those who reported $\geq$ 42 weeks of gestation).
Delivery mode	What is the mode of delivery of your child?	Vaginal delivery, Cesarean section
Pregnancy order	What's the order of this child's pregnancy? (including abortion, spontaneous abortion and normal pregnancy)	-
Delivery order	What's the order of this child's delivery?	-
Twin birth	Is your child one of the twins?	Yes, No
Birth length	What was your child's birth length?	Continuous (to the nearest 0.1 cm)
Birth weight	What was your child's birth weight?	Continuous (to the nearest 0.1 kg)
Infancy feeding	How did you feed your child for 6 months after birth?	Pure breastfeeding, Partial breastfeeding (both of breast milk and formula), Non-breastfeeding
Breastfeeding duration	Those who answered “Pure breastfeeding” or “Partial breastfeeding” were asked “How many months did you breastfeed your child? ”.	Continuous (to the nearest 1 month)
Time to introduce solid food	How many months old did you introduce solids to your child?	Continuous (to the nearest 1 month)

**Lifestyle-related Factors**		
Sedentary time	How much time is your child sedentary in total on a typical day? (including classes, watching TV, reading, studying, doing homework, etc.)	Continuous (to the nearest 1 minute)
Screen time	How much time does your child spend looking at electronic screens on a typical day? (e.g. mobile phones, TV, tablets, etc.)	Continuous (to the nearest 1 minute)
Outdoor activities	How much time does your child usually spend in outdoor physical activities on a typical day? (e.g. running in the playground, playing ball games, cycling, hiking, etc.)	Continuous (to the nearest 1 minute)
Bedtime	What time does your child usually go to bed at night?	Continuous (to the nearest 1 minute)
Eating speed	How much time does it take on average for your child to have each meal? (to the nearest 1 minute)	According to the existing literature, we divided meal times into three groups: (1) those who spent <15 minutes per meal; (2) those who spent ≥ 15 minutes to < 30 minutes per meal; and (3) those who spent $\geq$ 30 minutes per meal.
Fast food intake frequency	How often does your child have fast food or fried food? (e.g. French fries, fried chicken, pizza, etc.)	Every day, 3-5 times weekly, 1-2 times weekly, None or once in a while
Sweet food intake frequency	How often does your child have sweet food? (e.g. sugary drinks, sweets, cakes, chocolates, etc.)	Every day, 3-5 times weekly, 1-2 times weekly, None or once in a while
Night meal intake frequency	How often does your child have night meal?	Every day, 3-5 times weekly, 1-2 times weekly, None or once in a while

**Health Status**		
Food allergy	Does your child have a history of food allergies? (redness, swelling of the skin, rash, abdominal pain, etc. within 2 hours after eating a certain food)	Yes, No
Drug allergy	Is your child allergic to any medications? Or has your child ever had allergic symptoms such as itchy skin or rashes after taking a certain medication?	Yes, No
Dental caries	Does your child have dental caries? If so, how many?	-
Chronic diseases	Does your child have any chronic diseases? (e.g. chronic kidney disease, congenital heart disease, hypothyroidism, etc.)	Yes, No
**Family Information**		
Maternal reproductive age	We asked the current age of the child's mother and then subtracted the child's age from the mother's age to obtain the mother's age when the child was born.	Continuous (to the nearest 1 year)
Paternal reproductive age	We asked the current age of the child's father and then subtracted the child's age from the father's age to obtain the father's age when the child was born.	Continuous (to the nearest 1 year)
Maternal BMI	Based on the self-reported height and weight of the child's mother, BMI was calculated as weight in kilograms divided by height in meters squared.	Continuous (to the nearest 0.01 kg/m^2)
Paternal BMI	Based on the self-reported height and weight of the child's father, BMI was calculated as weight in kilograms divided by height in meters squared.	Continuous (to the nearest 0.01 kg/m^2)

Maternal education level	What is the education level of the child's mother?	High school degree or below, Bachelor's degree, Master's degree or above
Paternal education level	What is the education level of the child's father?	High school degree or below, Bachelor's degree, Master's degree or above
Family income	What is the gross annual income of the child's household? (yuan per year)	< 100,000, [100,000 , 300,000), ≥ 300,000

Table S2. The comparison of 3 ensemble learning algorithms adopted in this study

Ensemble learning algorithms	Advantages	Disadvantages
Decision tree	Decision tree can be visually illustrated and be easily understood.<br>There is no restriction on data sizes.<br>No specific distributions are assumed for independents, allowing the existence of collinearity and without transformation (such as max-min standardization).<br>Independent variables can be either continuous, categorical or factorial, and are less sensitive to extremes or outliers.<br>The training process is highly automated, and the importance ranking can be derived.	Decision tree is susceptible to underfitting and overfitting, and often insufficient in fitting process.<br>Decision tree is sensitivity to data, with a tiny change in training dataset leading to large changes in decision logistics.<br>In the case of more independent variables, decision tree is usually more complicated, and biases cannot be fully excluded with a large number of nodes. Sometimes, decision tree is counterintuitive.
Random forest	Random forest can handle collinear and noising dataset, and is suitable for a large number of features and extreme sample sizes.<br>Dependent variable and independent variables can be either continuous or categorical.<br>Only one key parameter is available for tuning, making the training process easier.<br>Random forest can be applied in clustering analyses for unsupervised learning processes.	Like decision tree, random forest is often hard to explain.<br>Fitting the random forest model might be time-consuming.
Gradient boosting machine	There is no need to standardize dataset before modelling, and gradient boosting machine is suitable for continuous or categorical dependent variable.<br>The construction of decision tree is made in a continuous way, and each tree tries to correct mistakes made by prior tree, with gradual improvement.<br>Gradient boosting machine is less susceptible to extremes or outliers.	The training process of parameter tuning is time-consuming and requires a significant amount of memory or processing power.<br>Gradient boosting machine is usually not suitable for high dimensional sparse data (the number of zeros is much larger than that of non-zeros in matrixes).<br>Gradient boosting machine has a large number of parameters for tuning, and the tuning process is based on experience and luck.

Table S3. Hyper-parameters of 3 ensemble learning algorithms adopted in this study

**Ensemble learning algorithms**	**Hyperparameters**
Decision tree	methods: anova, poisson, class or exp.<br>cp values: 0.01 to 0.1 by 0.01.<br>split methods: information or gini.
Random forest	number of trees: 100 to 10000 by 100.
Gradient boosting machine	total number of trees: 10 to 10000 by 10.<br>maximum depth of each tree: 1 to 10 by 1.<br>shrinkage parameter: 0.01 to 1.0 by 0.01.<br>cross-validation folds: 2 to 20 by 1.

Table S4. STROBE Statement—checklist of items that should be included in reports of observational studies

	Item No	Recommendation	Page No
**Title and abstract**	1	(*a*) Indicate the study's design with a commonly used term in the title or the abstract	1
		(*b*) Provide in the abstract an informative and balanced summary of what was done and what was found	2-3
**Introduction**			
Background/rationale	2	Explain the scientific background and rationale for the investigation being reported	4-5
Objectives	3	State specific objectives, including any prespecified hypotheses	5
**Methods**			
Study design	4	Present key elements of study design early in the paper	5-6
Setting	5	Describe the setting, locations, and relevant dates, including periods of recruitment, exposure, follow-up, and data collection	5-6
Participants	6	(*a*) *Cohort study*—Give the eligibility criteria, and the sources and methods of selection of participants. Describe methods of follow-up<br>*Case-control study*—Give the eligibility criteria, and the sources and methods of case ascertainment and control selection. Give the rationale for the choice of cases and controls<br>*Cross-sectional study*—Give the eligibility criteria, and the sources and methods of selection of participants	5-6
		(*b*) *Cohort study*—For matched studies, give matching criteria and number of exposed and unexposed<br>*Case-control study*—For matched studies, give matching criteria and the number of controls per case	NA
Variables	7	Clearly define all outcomes, exposures, predictors, potential confounders, and effect modifiers. Give diagnostic criteria, if applicable	6-7
Data sources/ measurement	8*	For each variable of interest, give sources of data and details of methods of assessment (measurement). Describe comparability of assessment methods if there is more than one group	6-7
6	9	Describe any efforts to address potential sources of bias	6
Study size	10	Explain how the study size was arrived at	6-7
Quantitative variables	11	Explain how quantitative variables were handled in the analyses. If applicable, describe which groupings were chosen and why	7
Statistical methods	12	(*a*) Describe all statistical methods, including those used to control for confounding	8-9
		(*b*) Describe any methods used to examine subgroups and interactions	NA
		(*c*) Explain how missing data were addressed	8
		(*d*) *Cohort study*—If applicable, explain how loss to follow-up was addressed<br>*Case-control study*—If applicable, explain how matching of cases and controls was addressed<br>*Cross-sectional study*—If applicable, describe analytical methods taking account of sampling strategy	8
		(*e*) Describe any sensitivity analyses	NA

Continued on next page

Results

Participants	13*	(a) Report numbers of individuals at each stage of study—eg numbers potentially eligible, examined for eligibility, confirmed eligible, included in the study, completing follow-up, and analysed	9-10
		(b) Give reasons for non-participation at each stage	NA
		(c) Consider use of a flow diagram	6
Descriptive data	14*	(a) Give characteristics of study participants (eg demographic, clinical, social) and information on exposures and potential confounders	10
		(b) Indicate number of participants with missing data for each variable of interest	NA
		(c) *Cohort study*—Summarise follow-up time (eg, average and total amount)	NA
Outcome data	15*	*Cohort study*—Report numbers of outcome events or summary measures over time	NA
		Case-control study—Report numbers in each exposure category, or summary measures of exposure	NA
		Cross-sectional study—Report numbers of outcome events or summary measures	10
Main results	16	(*a*) Give unadjusted estimates and, if applicable, confounder-adjusted estimates and their precision (eg, 95% confidence interval). Make clear which confounders were adjusted for and why they were included	10-12
		(*b*) Report category boundaries when continuous variables were categorized	10-12
		(*c*) If relevant, consider translating estimates of relative risk into absolute risk for a meaningful time period	NA
Other analyses	17	Report other analyses done—eg analyses of subgroups and interactions, and sensitivity analyses	NA

Discussion

Key results	18	Summarise key results with reference to study objectives	12
Limitations	19	Discuss limitations of the study, taking into account sources of potential bias or imprecision. Discuss both direction and magnitude of any potential bias	16
Interpretation	20	Give a cautious overall interpretation of results considering objectives, limitations, multiplicity of analyses, results from similar studies, and other relevant evidence	12-16
Generalisability	21	Discuss the generalisability (external validity) of the study results	16

Other information

Funding	22	Give the source of funding and the role of the funders for the present study and, if applicable, for the original study on which the present article is based	17

*Give information separately for cases and controls in case-control studies and, if applicable, for exposed and unexposed groups in cohort and cross-sectional studies.

Note: An Explanation and Elaboration article discusses each checklist item and gives methodological background and published examples of transparent reporting. The STROBE checklist is best used in conjunction with this article (freely available on the Web sites of PLoS Medicine at http://www.plosmedicine.org/, Annals of Internal Medicine at http://www.annals.org/, and Epidemiology at http://www.epidem.com/). Information on the STROBE Initiative is available at www.strobe-statement.org.

Figure S1. Selection flowchart of study participants

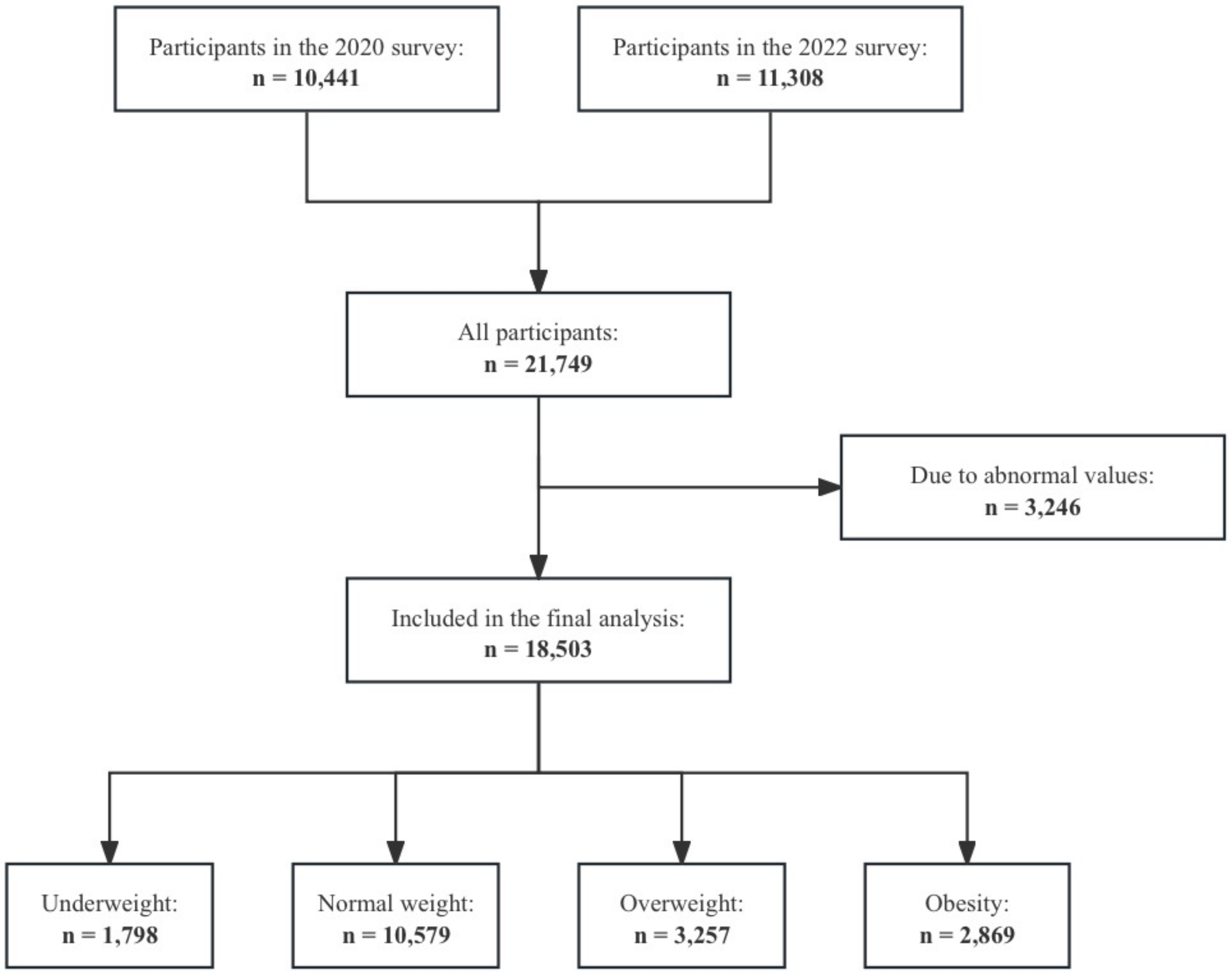

Figure S2. Decision curve analysis of 3 machine learning algorithms annexed with Logistic regression for childhood weight status

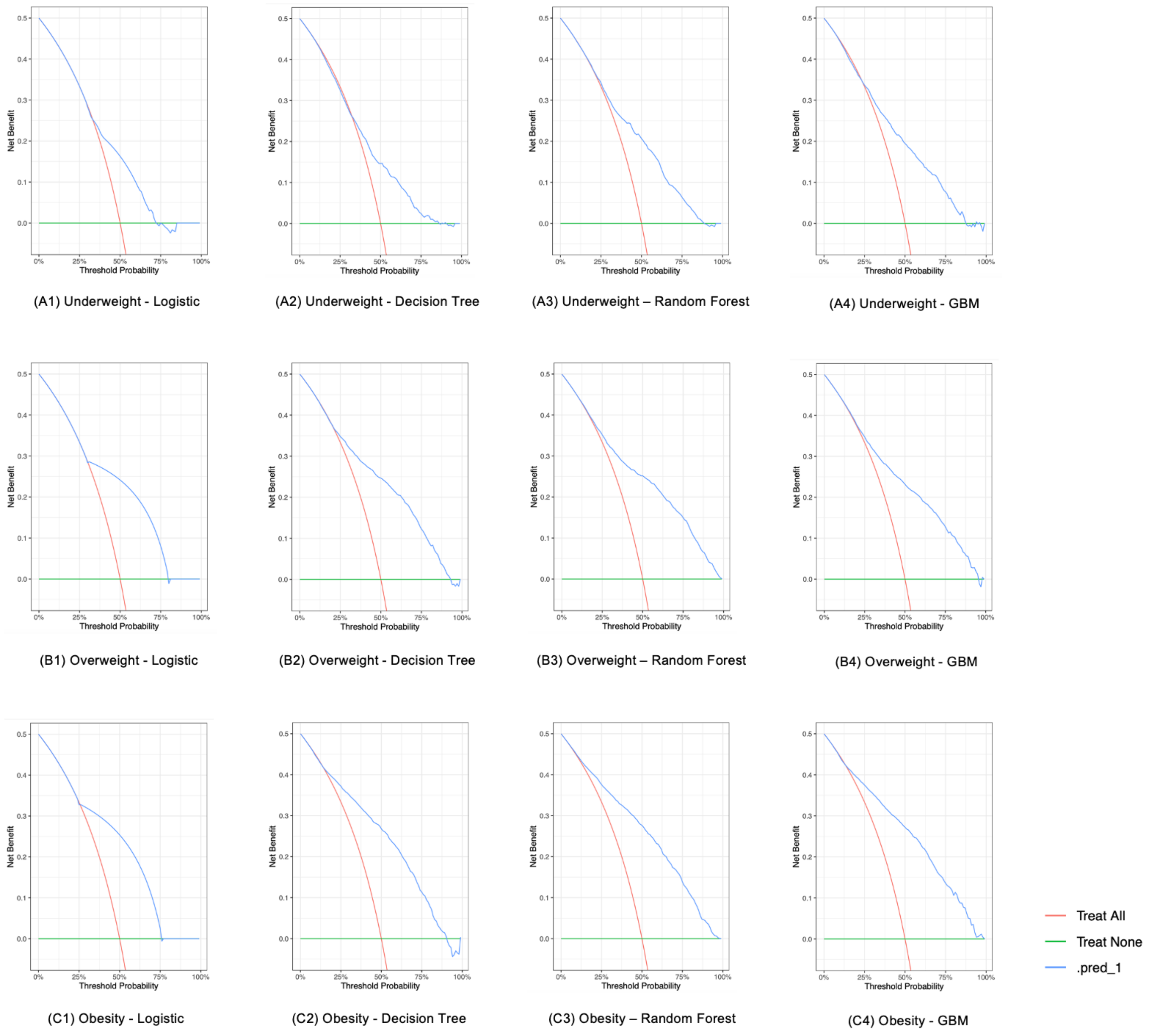

Abbreviations: GBM, Gradient Boosting Machine.

*Weight status was defined according to the criteria recommended by the World Health Organization (WHO).
